# Impact of Ca^2+^-Induced PI(4,5)P_2_ Clusters on PH-YFP Organization and Protein-Protein Interactions

**DOI:** 10.3390/biom12070912

**Published:** 2022-06-29

**Authors:** Luís Borges-Araújo, Marina E. Monteiro, Dalila Mil-Homens, Nuno Bernardes, Maria J. Sarmento, Ana Coutinho, Manuel Prieto, Fábio Fernandes

**Affiliations:** 1 IBB—Institute for Bioengineering and Biosciences, Instituto Superior Técnico, University of Lisbon, 1049-001 Lisbon, Portugal; lpborgesaraujo@tecnico.ulisboa.pt (L.B.-A.); dalilamil-homens@tecnico.ulisboa.pt (D.M.-H.); nuno.bernardes@tecnico.ulisboa.pt (N.B.); ana.coutinho@tecnico.ulisboa.pt (A.C.); manuel.prieto@tecnico.ulisboa.pt (M.P.); 2 Associate Laboratory i4HB—Institute for Health and Bioeconomy at Instituto Superior Técnico, University of Lisbon, 1049-001 Lisbon, Portugal; 3 Centro de Química-Física Molecular and Institute of Nanoscience and Nanotechnology, Instituto Superior Técnico, University of Lisbon, 1049-001 Lisbon, Portugal; marina.monteiro@ist.utl.pt (M.E.M.); maria.sarmento@medicina.ulisboa.pt (M.J.S.); 4 Instituto de Medicina Molecular, Faculty of Medicine, University of Lisbon, Avenida Prof. Egas Moniz, 1649-028 Lisbon, Portugal; 5 Departamento de Química e Bioquímica, Faculty of Sciences, University of Lisbon, 1749-016 Lisbon, Portugal; 6 Department of Bioengineering, Instituto Superior Técnico, University of Lisbon, 1049-001 Lisbon, Portugal

**Keywords:** PI(4,5)P_2_, nanodomains, Ca^2+^, PI(4,5)P_2_-binding proteins

## Abstract

Despite its low abundance, phosphatidylinositol 4,5-bisphosphate (PI(4,5)P_2_) is a key modulator of membrane-associated signaling events in eukaryotic cells. Temporal and spatial regulation of PI(4,5)P_2_ concentration can achieve localized increases in the levels of this lipid, which are crucial for the activation or recruitment of peripheral proteins to the plasma membrane. The recent observation of the dramatic impact of physiological divalent cation concentrations on PI(4,5)P_2_ clustering, suggests that protein anchoring to the plasma membrane through PI(4,5)P_2_ is likely not defined solely by a simple (monomeric PI(4,5)P_2_)/(protein bound PI(4,5)P_2_) equilibrium, but instead depends on complex protein interactions with PI(4,5)P_2_ clusters. The insertion of PI(4,5)P_2_-binding proteins within these clusters can putatively modulate protein–protein interactions in the membrane, but the relevance of such effects is largely unknown. In this work, we characterized the impact of Ca^2+^ on the organization and protein–protein interactions of PI(4,5)P_2_-binding proteins. We show that, in giant unilamellar vesicles presenting PI(4,5)P_2_, the membrane diffusion properties of pleckstrin homology (PH) domains tagged with a yellow fluorescent protein (YFP) are affected by the presence of Ca^2+^, suggesting direct interactions between the protein and PI(4,5)P_2_ clusters. Importantly, PH-YFP is found to dimerize in the membrane in the absence of Ca^2+^. This oligomerization is inhibited in the presence of physiological concentrations of the divalent cation. These results confirm that cation-dependent PI(4,5)P_2_ clustering promotes interactions between PI(4,5)P_2_-binding proteins and has the potential to dramatically influence the organization and downstream interactions of PI(4,5)P_2_-binding proteins in the plasma membrane.

## 1. Introduction

The binding of peripheral proteins to biological membranes is known to be a fundamental process in cell function and homeostasis, not just by modulating local membrane dynamics, but also by organizing and regulating protein signaling complexes. In this context, lipid–protein interactions play a crucial role in the correct recruitment and organization of peripheral proteins by establishing interactions through their headgroups and hydrocarbon chains with protein membrane-binding domains [1]. This process becomes increasingly convoluted when we consider not only the large variety of peripheral binding proteins with distinct binding mechanisms, but also the variable and remarkably complex lipidome of biological membranes. This variability opens the way for the formation of membranes with distinct biophysical properties that can influence both protein-lipid and downstream protein-protein interactions. Overall, peripheral protein membrane binding is a complex process that is spatially and temporally regulated, and which is still not fully understood.

Phosphatidylinositol 4,5-bisphosphate (PI(4,5)P_2_) is the most abundant polyphosphoinositide found in the inner leaflet of the plasma membrane of mammalian cells, comprising around 1 mol.% of the total membrane phospholipids [2,3]. The two phosphorylations at positions 4 and 5 of the inositol headgroup give rise to a larger-than-average headgroup, with a highly negative charge density that stands out in the inner leaflet of the plasma membrane [4]. PI(4,5)P_2_ is known to exhibit compartmentalization at the plasma membrane [5,6,7]. These PI(4,5)P_2_-enriched domains are most likely associated with specific of PI(4,5)P_2_–protein [8] and PI(4,5)P_2_–cation interactions. In fact, studies indicate that as much as two-thirds of PI(4,5)P_2_ in the inner leaflet of the plasma membrane is somehow sequestered [6]. Recent results from our laboratory showed that the actin cytoskeleton was particularly important for lateral segregation of PI(4,5)P_2_ in the plasma membrane of HeLa cells [5]. As a result of these distinct biophysical characteristics [9], PI(4,5)P_2_ plays a critical role in membrane lipid–protein interactions, being responsible for the targeting of several protein domains to the plasma membrane, as well as spatially and temporally regulating their activity [10]. In fact, PI(4,5)P_2_ has been reported to be associated with numerous vital cell functions such as cell adhesion and motility [11], vesicle endocytosis [12,13,14], exocytosis [13,15,16], and ion channel transport [17]. There have been several PI(4,5)P_2_-binding domains identified, including the PX domains [18,19,20], C2 domains [21], ENTH domains [22], Tubby domains [23], and most notably the PH domains [24,25].

The PH domain is a 100–120 residue long protein domain found in numerous proteins involved in a multitude of cellular signaling processes. Initially, it was thought that most PH domains directed membrane targeting of their host proteins by binding to phosphoinositides (PIs). However, it is now known that, of the total number of PH domains identified, only a small minority bind to phospholipids. In fact, few PH domains bind specifically to PIs [26]. Despite this, the isolated PLCδ1 PH domain, in particular, was found to bind with high affinity and specificity to PI(4,5)P_2_ and its soluble headgroup, inositol trisphosphate (IP_3_) [27]. Studies of PI(4,5)P_2_ binding by the PLCδ1 PH domain provided the first demonstrations of specific PI recognition by a PH domain and provided the foundations for how binding domains recognize specific PIs in cellular membranes [28]. Indeed, PLCδ1 PH domains are still to this day used as excellent protein models to study PI(4,5)P_2_–protein interactions and PI(4,5)P_2_ organization. PH domains are electrostatically polarized with the positively charged end coinciding with the three variable loops that have been suggested to be the phosphorylated inositol-binding site [26]. It is through this region that these domains bind to the polyphosphorylated inositol ring [29] via direct hydrogen bonds with seven amino-acid residues that bury the 4- and 5-phosphate groups in the binding pocket. These interactions suggest that stereochemical cooperativity enhances specificity [30].

Recently, there have been a plethora of studies focused on the interactions between PI(4,5)P_2_ and divalent cations. PI(4,5)P_2_ has been shown to establish strong electrostatic interactions between its negatively charged headgroup and divalent cations, especially with Ca^2+^. In mammalian cells, Ca^2+^ is a common secondary messenger with an important role in signal transduction, typically binding and regulating proteins directly [31]. When Ca^2+^ interacts with PI(4,5)P_2_, it alters its lateral organization and induces its segregation into nanodomains [32,33,34,35]. This has been demonstrated in fully physiological conditions, through the incorporation of PI(4,5)P_2_ fluorescent analogues on PI(4,5)P_2_ clusters in free standing lipid bilayers using fluorescence spectroscopy methodologies [32,36,37,38]. The segregation of PI(4,5)P_2_ into these nanodomains is likely to hinder the dynamics of bound peripheral proteins, as well as their downstream interactions. Furthermore, Ca^2+^ has also been shown to influence both PI(4,5)P_2_ electrostatics [39] and headgroup conformation [40], via molecular interactions with the three phosphate groups. These effects can also substantially impact PI(4,5)P_2_–protein interactions.

Several PI(4,5)P_2_-binding proteins are known to be sensitive to variations in Ca^2+^ concentration [10]. In most of these cases, Ca^2+^ causes changes in protein folding/electrostatics that modulate the affinity of PI(4,5)P_2_-binding proteins. In these proteins, PI(4,5)P_2_ and Ca^2+^ binding typically occur at different binding sites [10]. However, the impact of the direct interaction of Ca^2+^ with PI(4,5)P_2_ on binding proteins has been given little attention. It is worth noting that the formation of PI(4,5)P_2_ clusters due to divalent cation crosslinking or the occurrence of the charge shielding effect, as described previously, are likely to have a dramatic effect not only on protein targeting or activity but also on the promotion/inhibition of downstream protein–protein interactions. Overall, many of the effects associated with cations and cation-induced nanodomains are yet to be fully understood, especially the extent to which they may impact PI(4,5)P_2_-dependent function and signalling. 

In this work, we aim to study how Ca^2+^-induced PI(4,5)P_2_ effects may influence the organization of PI(4,5)P_2_-binding proteins, using the PH-YFP protein construct as a model. PH-YFP is a fusion protein that contains an isolated PLCδ1 PH domain linked to a YFP variant that lacks the A206K mutation at the protein dimerization interface and is, thus, prone to dimerization [41]. The results presented here show, through a complementary set of fluorescence techniques, that Ca^2+^ within its physiological range of intracellular concentrations can modulate downstream protein–protein interactions of PI(4,5)P_2_-binding proteins. We show that PH-YFP membrane diffusion rates are dominated by the diffusion of Ca^2+^-induced PI(4,5)P_2_ clusters, asserting that PH-YFP remains associated to PI(4,5)P_2_ clusters and is unable to sequester a lipid from these structures. This indicates that these clusters have the potential to influence peripheral protein dynamics. Moreover, we show that Ca^2+^, in general, disrupted PH-YFP oligomerization in a reversible manner, which was dependent on its concentration. We hypothesize that this disruption could be the associated with increasing PI(4,5)P_2_ cluster sizes.

## 2. Materials and Methods

### 2.1. Materials

1-Palmitoyl-2-oleoyl-*sn*-glicero-3-phosphocholine (POPC), 1,2-dioleoyl-*sn*-glycero-3-phospho-(1′-myo-inositol-4′,5′-bisphosphate) (PI(4,5)P_2_), and 1,2-dioleoyl-*sn*-glycero-3-phosphoethanolamine-*N*-(cap biotinyl) (biotinylated DOPE) were purchased from Avanti Polar Lipids (Alabaster, AL, USA). Lipid stock solutions were prepared in chloroform with the exception of the PIs that were prepared in chloroform and methanol (MeOH) (2:1 vol/vol). Both solvents were obtained from Merck (Darmstadt, Germany) and were of spectroscopic grade. Phosphate-buffered saline (PBS), 4-(2-hydroxyethyl)-1-piperazineethanesulfonic acid (HEPES), ethanol (EtOH), NaCl, sucrose, glucose, CaCl_2_, imidazole, glycerol, bovine serum albumin (BSA), BSA-biotin, and avidin were purchased from Sigma-Aldrich. Rhodamine 110 and Fluo-5N were obtained from Molecular Probes, Invitrogen (Eugene, OR, USA).

### 2.2. PH-YFP Expression and Purification

PH-YFPxpET28a was made from YFP(d)-PHxpCDNA3 [42], which was a gift from Dr. Kees Jalink, (Division of Cell Biology, Netherlands Cancer). Briefly, the PH-YFP sequence flanked by BamHI and NotI restriction sites was inserted into a pET28a vector. PH-YFPxpet28a was expressed in *Escherichia coli* BL21 (DE). The cells were transformed by electroporation and incubated in LB agar plates with kanamycin overnight at 37 °C. A pre-inoculum was made with a single colony from the plate and incubated overnight. The appropriate pre-inoculum volume was added for an initial OD_600 nm_ of 0.1 in 100 mL LB medium containing kanamycin. The culture was incubated at 24 °C, 250 rpm, until the OD_600 nm_ reached approximately 0.6–0.8. Expression was then induced with 0.2 mM isopropyl β-d-1-thiogalactopyranoside (IPTG) at 24 °C and 250 rpm for 3 h. Cells were harvested from the culture by centrifugation (6000× *g* for 15 min) at 4 °C. The pellet was suspended in lysis buffer (50 mM PBS, 300 mM NaCl, pH 8.0) with an added protease inhibitor cocktail and sonicated until complete DNA fragmentation was observed. Specifically, 10 × 15 s bursts with a 15 s cooling period in between were performed. The lysate was centrifuged (17,600× *g* for 5 min) at 4 °C to remove cellular debris. The supernatant was transferred to a clean tube without disturbing the pellet and centrifuged again at 17,600× *g* for 1 h at 4 °C. PH-YFP was then purified making use of its histidine tag using Protino Ni-TED 2000 packed columns (Macherey-Nagel, Düren, Germany). Cleared lysates were applied to the column and washed with eight column volumes of lysis buffer. The polyhistidine-tagged protein eluted with five column volumes of elution buffer (50 mM PBS, 300 mM NaCl, 250 mM imidazole, pH 8.0). Here, 3 mL fractions were collected by gravity flow and monitored for protein presence by UV 280 nm absorption. Most of the eluted protein could be found in the first fractions. SDS-PAGE analysis was used to determine protein purity. The purified protein was stored in a 10 mM PBS buffer containing 140 mM NaCl and 10% glycerol at pH 7.4.

### 2.3. Liposome Preparation

Large unilamellar vesicles (LUVs) were prepared by extrusion of multilamellar vesicles [43]. Briefly, the lipid mixtures were prepared from phospholipid stock solutions, dried under a nitrogen flux, and left in vacuum for 3 h to remove traces of solvent. Multilamellar vesicles were then obtained through the solubilization of the lipid films in the appropriate experimental buffer. Freeze–thaw cycles were performed, using liquid nitrogen and a water bath typically set to 60 °C. The thawing temperature used was always higher than the melting temperature of the lipid with the highest melting temperature in the mixture, to re-equilibrate and homogenize the samples. LUVs were obtained by extrusion at room temperature, using an Avanti Mini-Extruder (Alabaster, AL, USA) and 100 nm pore size polycarbonate membranes (Whatman, Buckinghamshire, UK). Typically, at least 21 passages through the extruder were performed.

Giant unilamellar vesicles (GUVs) were obtained by gel-assisted formation, according to a method previously described [44]. The lipid mixtures were prepared from stock solutions in chloroform to a final concentration of 1.5 mM. For the PH-YFP experiments, the mixtures were composed of 95% POPC and 5% PI(4,5)P_2_. DOPE-Cap-biotin was included in the mixture at a biotinylated lipid/total lipid ratio of 1/750,000 [45]. A solution of 5% (*w*/*w*) polyvinyl alcohol (PVA) MW ~145,000 and 280 mM of sucrose was spread in a μ-slide chamber from Ibidi (Munich, Germany) and left to dry for 15 min at 50 °C. The desired lipid mixture was then spread on the PVA surface. The solvent was evaporated for 15 min under vacuum. After evaporation of the solvent, the appropriate buffer solution was added, allowing for GUV formation for 60 min at room temperature. After the formation, GUVs were transferred to a μ-slide chamber with the appropriate coating and left to rest for 10 min to allow for GUV deposition and immobilization. In order to immobilize the GUVs through the interaction with the biotinylated lipid and minimize nonspecific protein adsorption to the surface, a mixed coating of BSA + BSA-biotin (9:1 molar ratio) and avidin was used. Failure to perform this passivation of the coverslip surface always resulted in significant PH-YFP adsorption. Ibidi μ-slide chambers were coated with 300 μL of a 0.9 mg/mL BSA and 0.1 mg/mL BSA-biotin mixture for 1 h. Afterward, the chambers were washed with filtered milliQ water and covered with a second layer of 300 μL of 0.01 mg/mL Avidin for 1 h. BSA, BSA-biotin and avidin solutions were prepared with milliQ water. Before adding the GUV solution, the chambers were washed with filtered milliQ water.

Buffer Ca^2+^ concentrations in the micromolar range were determined using the fluorescent calcium indicator Fluo-5N pentapotassium salt, following the instructions of the manufacturer.

### 2.4. Steady-State Fluorescence Spectroscopy

Fluorescence measurements were carried out with a SLM-Aminco 8100 Series 2 spectrofluorimeter (Rochester, New York, NY, USA) with double excitation and emission monochromators (MC-400), in right-angle geometry. The light source was a 450 W Xe arc lamp and the reference was a Rhodamine B quantum counter solution. Quartz cuvettes (0.5 × 0.5 cm) from Hellma Analytics were used. Temperature was controlled to 25 °C. Polarization of excitation and emission light was obtained by specific rotation of Glan-Thompson polarizers. Blank subtraction was taken into account in all fluorescence intensity and anisotropy measurements.

Steady-state fluorescence anisotropy, 〈r〉, is defined as follows [46]:

(1) $$\langle r\rangle =\frac{I_{VV}-G*I_{VH}}{I_{VV}+2*G*I_{VH}}\;;\;G=\frac{I_{HV}}{I_{HH}},$$

where $I_{Vj}$ represents the steady-state vertical (parallel, $I_{VV}$) and horizontal (perpendicular, $I_{VH}$) components of the fluorescence emission with vertically polarized excitation. The *G* factor is measured using the vertical ($I_{HV}$) and horizontal ($I_{HH})$ components of the fluorescence emission with horizontaly polarized excitation. 

PH-YFP partition to LUVs was followed by steady-state fluorescence intensity and anisotropy measurements, by exploiting the fluorescence emission of the attached YFP protein. PH-YFP membrane interactions with LUVs were studied in the presence of different lipid concentrations (up to 100 μM) and in the presence and absence of Ca^2+^. LUVs used in these experiments were composed of POPC and varying molar ratios of (18:1) PI(4,5)P_2_ (99:1, 97:3 and 95:5 molar ratios). Then, 100 nM of PH-YFP was incubated for 5 min at 37 °C with the different lipid concentrations. The experimental buffer used for the PH-YFP experiments consisted of 10 mM Na_2_HPO_4_, 140 mM NaCl at pH 7.4. Furthermore, 5 mM of EDTA was included in the experiment buffer to study the interactions in the absence of Ca^2+^. To study the influence of Ca^2+^, 100 μM of CaCl_2_ was included in the experimental buffer. For each sample, three independent replicates were measured.

#### Photobleaching Assay

Photobleaching of protein and liposome samples was performed using a Xe (450 W) light source, focused onto the sample via a magnifying glass. The samples were exposed to the light source for varying durations, in order to obtain different relative photobleaching percentages. Fluorescence anisotropy was then measured as previously described. We could then fit a model that predicts the formation of oligomers to the obtained rate at which fluorescence anisotropy recovered with photobleaching percentage and attempt to determine the PH-YFP oligomerization number. This model is based on a binomial distribution, where it predicts the different fractions of still fluorescent oligomers for a given oligomerization number and photobleaching percentage.

### 2.5. Confocal Fluorescence Microscopy

Confocal laser scanning fluorescence microscopy measurements were performed on a Leica TCS SP5 (Leica Microsystems CMS GmbH, Manheim, Germany) inverted confocal microscope (DMI600). Excitation lines provided by an argon laser were focused into the sample through an apochromatic water immersion objective (63×, NA 1.2; Zeiss, Jena, Germany). A 111.4 μm diameter pinhole in front of the image plane blocked out-of-focus signal. Images were acquired at 100 Hz, exciting PH-YFP at 488 nm and collecting emission between 500 and 600 nm. For each GUV, an image was taken roughly at the equatorial plane. Membrane fluorescence and GUV radius were automatically quantified making use of a homemade MATLAB script. No correlation between GUV radius and membrane fluorescence was found.

### 2.6. Fluorescence Fluctuation Spectroscopy

FFS measurements were carried out using the same optical path described for the confocal imaging, except that fluorescence emission was detected using avalanche photodiodes (APDs) after passing through a 500–550 bandpass filter. Excitation of PH-YFP was performed with the 488 nm Ar laser line. For samples in solution, the focal volume was focused ~100 μm above the top surface of the cover slide, and five autocorrelation curves were sequentially obtained with an acquisition time of 60 s at a 500 kHz sampling frequency. At least four independent samples were measured per condition. Fluorescence fluctuations from GUVs with bound fluorescent proteins were recorded from the top of the vesicle, with the focal volume centered in the focal plane with maximum fluorescence intensity. Five autocorrelation curves were sequentially obtained for each sample with an acquisition time of 20 s at a 100 kHz sampling frequency. At least 3–10 GUVs were measured per condition. Specific theoretical background behind the fluorescence correlation spectroscopy (FCS) and photon counting histogram analysis (PCH) models and methods is available in the Supplementary Materials.

### 2.7. Statistical Analysis

Statistical analysis was performed for steady-state fluorescence anisotropy results using regular two-way analysis of variance tests. The two factors accounted for were lipid concentration and whether the sample was in the presence or absence of calcium. F-statistics, degrees of freedom, and *p*-values are reported in the manuscript as called for. No post hoc comparisons were performed. Statistical analysis of FFS results was performed using Mann–Whitney U tests. Sample sizes and *p*-values are reported in the manuscript as called for. Statistical analysis was performed using GraphPad Prism 7.00, GraphPad Software, La Jolla, CA, USA.

## 3. Results

In this work, we were interested in understanding whether Ca^2+^ and Ca^2+^-induced PI(4,5)P_2_ clustering could directly influence peripheral protein–protein interactions (in this case, PH-YFP membrane organization and oligomerization). PH-YFP is a fusion protein that contains an isolated PLCδ1 PH domain linked to a yellow fluorescent protein variant, YFP. Apart from the PI(4,5)P_2_-binding surface that faces the membrane, the surface of the PH domain is fairly negatively charged, and no tendency for oligomerization has been reported. On the other hand, the YFP variant used (lacking the A206K mutation) is a weak dimer [47], which dimerizes when present at high concentrations. As a result, the PH-YFP construct, also showed a tendency to oligomerize, especially when bound to the membrane due to the local concentration effect. PH-YFP is, thus, an excellent protein model for the study of protein–protein and protein–lipid interactions of PI(4,5)P_2_-binding proteins.

### 3.1. Homo-FRET Assays

The fluorescence anisotropy of PH-YFP in solution was 0.312 (in the absence of lipids), which is consistent with a monomeric state of the protein as expected [48]. As the value of fluorescence anisotropy of soluble PH-YFP is already quite high, the reduced dynamics introduced by membrane binding is expected to have only a very moderate impact on fluorescence anisotropy of the monomer. On the other hand, oligomerization of the protein in the membrane would result in Förster resonance energy transfer (homo-FRET) between interacting YFP tags and increased fluorescence depolarization. This would then result in a decrease in fluorescence anisotropy values.

**Figure 1:**
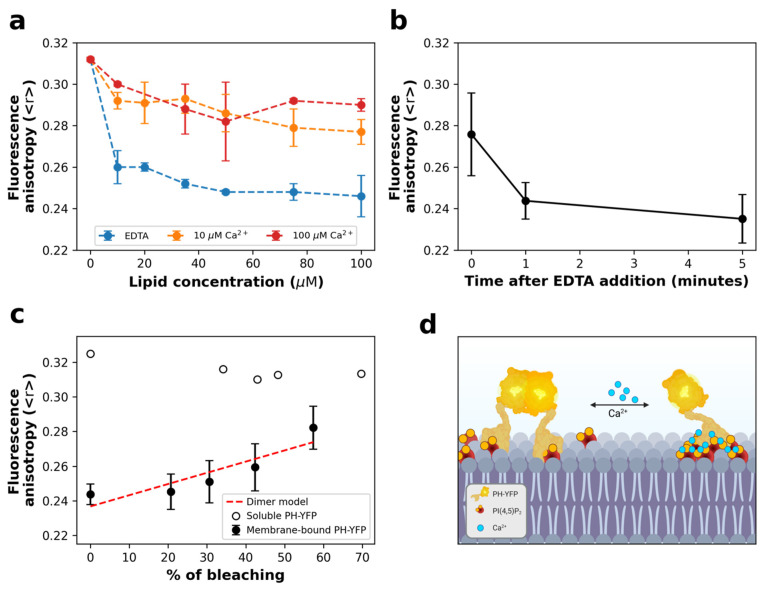
PH-YFP partitions to PI(4,5)P_2_ containing LUVs and undergoes significant oligomerization in the absence of Ca^2+^. (**a**) PH-YFP partition to POPC:PI(4,5)P_2_ (95:5 molar ratio) LUVs, followed through changes in steady-state fluorescence anisotropy, in the presence of 10 μM Ca^2+^ (orange), 100 μM Ca^2+^ (red), and 5 mM EDTA (blue). Dashed lines are just a guide to the eye. (**b**) Kinetics of fluorescence anisotropy response to the addition of 5 mM EDTA to PH-YFP and PI(4,5)P_2_ containing LUVs in the presence of 100 μM Ca^2+^. Total lipid concentration was 100 µM. (**c**) Photobleaching assay of 100 μM PH-YFP, both in solution (empty circles) and incubated with POPC:PI(4,5)P_2_ (95:5 molar ratio) LUVs, in the presence of 5 mM EDTA (full circles). (**d**) Illustration of PI(4,5)P_2_-bound PH-YFP dimerization modulation by Ca^2+^. Created with BioRender.com. Values represent means ± standard deviations. Values for each condition are averages of three different independent replicates.

We started by following PH-YFP partition to POPC:PI(4,5)P_2_ (95:5 molar ratio) LUVs in the presence of different Ca^2+^ concentrations. (Figure 1a). In general, we observed a decrease in YFP fluorescence anisotropy with increasing total lipid concentration, confirming that oligomerization took place upon membrane binding. Comparing the results with different Ca^2+^ concentrations, PH-YFP presented a significantly lower anisotropy in the absence of Ca^2+^ than when compared to the results obtained in the presence of 10 μM (F(1,24) = 199.5, *p* < 0.0001) and 100 μM Ca^2+^ (F(1,24) = 167.3, *p* < 0.0001). These results appear to suggest that the formation of Ca^2+^-induced PI(4,5)P_2_ clusters could disrupt YFP oligomerization in the membrane to some extent. When 5 mM EDTA was added to PH-YFP previously incubated with 100 μM LUVs containing POPC:PI(4,5)P_2_ (95:5 molar ratio) in 100 μM Ca^2+^ buffer (Figure 1b), we observed that fluorescence anisotropies decreased from the values observed in the presence of Ca^2+^ to roughly those observed in the absence of the cation. This result hints that the inhibition of PH-YFP oligomerization through the formation of Ca^2+^-induced PI(4,5)P_2_ clusters is reversible. This observation has important implications as Ca^2+^-induced clustering could act as a toggle switch to modulate interactions between PI(4,5)P_2_-binding proteins in the plasma membrane. Calcium levels did not result in inhibition of membrane association by PH-YFP, as full membrane association was confirmed for all conditions at 100 μM lipid (Supplementary Figure S1).

To confirm that these variations in fluorescence anisotropy were due to PH-YFP oligomerization and not due to changes in binding efficiency, we performed a photobleaching assay (Figure 1c). In this assay, samples were exposed to intense light for varying amounts of time to obtain different YFP photobleaching efficiencies. While photobleaching of PH-YFP in solution did not cause any significant change in fluorescence anisotropy, photobleaching of PH-YFP samples incubated with 100 μM of PI(4,5)P_2_ containing LUVs, in the absence of Ca^2+^, led to the progressive recovery of fluorescence anisotropy values, reaching monomer anisotropy values at high photobleaching rates. This confirms that the decrease in fluorescence anisotropy observed for the samples in the absence of Ca^2+^ occurred due to homo-FRET within PH-YFP oligomers. The rate at which the fluorescence anisotropy recovered with photobleaching percentage could be satisfactorily fitted using a model that predicts the formation of dimers. However, this does not exclude the possibility of formation of higher-order oligomers, as the quenching data could also be fitted using a model that predicts the formation of trimers (although with a slightly lower accuracy). 

### 3.2. Fluorescence Fluctuation Spectroscopy (FFS) Assays

**Figure 2:**
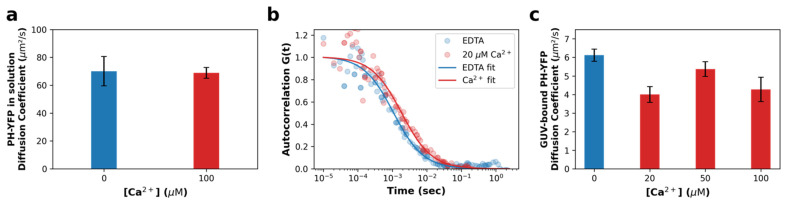
PH-YFP is unable to sequester lipids from Ca^2+^-induced PI(4,5)P_2_ clusters. (**a**) Diffusion coefficients obtained for PH-YFP in solution, in the presence (red) and absence (blue) of Ca^2+^. (**b**) Representative FCS autocorrelation curves of PH-YFP in POPC:PI(4,5)P_2_ (95:5 molar ratio) GUVs, in the presence and absence of Ca^2+^. (**c**) Diffusion coefficients obtained for PH-YFP in POPC:PI(4,5)P_2_ (95:5 molar ratio) GUVs, in the presence and absence of Ca^2+^. Values represent means ± standard errors. Values from solution measurements for each condition are averages of five independent samples. Values from GUV measurements for each condition are averages of at least five different GUVs ($N_{0\;\mu M\;Ca^{2+}}=10,\;N_{20\;\mu M\;Ca^{2+}}=8,\;N_{50\;\mu M\;Ca^{2+}}=5,\;N_{100\;\mu M\;Ca^{2+}}=5$).

For Ca^2+^-induced PI(4,5)P_2_ clusters to be able to influence downstream PH-YFP oligomerization, we would expect PLCδ1-PH domains to be unable to sequester bound PI(4,5)P_2_ from within the clusters, so that the protein remains anchored to these structures. To confirm that this occurs, fluorescence correlation spectroscopy (FCS) measurements were carried out in giant unilamellar vesicles (GUVs). When analyzing the free diffusion of PH-YFP in solution (Figure 2a), in the presence and absence of Ca^2+^, both sets of autocorrelation data were properly fitted to a single component 3D diffusion model and did not require a second component to be introduced to the analysis, suggesting a single population of diffusing species, as expected. We report that Ca^2+^ does not alter the diffusion of the protein in solution (*p* > 0.999 according to the Mann–Whitney U test), which presented a diffusion coefficient in the absence and presence of Ca^2+^ of D = 70.09 ± 4.72 μm^2^·s^−1^ and D = 68.87 ± 1.74 μm^2^·s^−1^, respectively, confirming that Ca^2+^ does not impact the PH-YFP oligomerization state before membrane binding.

We followed by studying PH-YFP diffusion in GUVs in response to the same conditions (Figure 2b,c). Autocorrelation datasets were also properly fitted to a single-component 2D diffusion model, not requiring a second component be introduced to the analysis, suggesting once more a single population of diffusing species (membrane-bound protein). Representative curves are presented in Figure 2b. Analyzing the diffusion data (Figure 2c), one can conclude that PH-YFP membrane diffusion appears to be dominated by PI(4,5)P_2_ diffusion at this protein-to-lipid ratio, as the values observed (D = 6.12 ± 0.32 μm^2^·s^−1^ (D + SEM) for the samples in the absence of Ca^2+^) are very similar to those reported by FCS for fluorescent analogues of PI(4,5)P_2_ in POPC GUVs (D = 8.01 ± 0.13 μm^2^·s^−1^ (D + SEM) for the samples in the absence of Ca^2+^) [32]. This suggests that the diffusion of the protein–lipid complex is dominated by the viscosity experienced by the PI(4,5)P_2_ inserted in the membrane, rather than the water-exposed protein, as has been reported for other PH domains [49]. Looking at the effect of Ca^2+^, the diffusion coefficient of PH-YFP decreased to some extent, going from D = 6.12 ± 0.32 μm^2^·s^−1^ (D + SEM) in the absence of Ca^2+^ to D = 4.27 ± 0.65 μm^2^·s^−1^ at 100 μM Ca^2+^ (*p* = 0.0193 according to the Mann–Whitney U test). This effect was observed even at 20 μM Ca^2+^ (D = 4.00 ± 0.42 μm^2^·s^−1^) (*p* = 0.002 according to the Mann–Whitney U test). This 30% decrease in PH-YFP diffusion in response to an increase in Ca^2+^ concentration is similar to that previously reported for a PI(4,5)P_2_ fluorescent analogue in GUVs with Ca^2+^ [32]. Altogether, these results clearly confirm that PH-YFP domains, after interaction with Ca^2+^-induced PI(4,5)P_2_ clusters, are not able to sequester the phospholipid from these PI(4,5)P_2_-enriched nanodomains and remain associated to these structures, with restricted lateral diffusion. This result confirms that Ca^2+^-induced nanodomains could potentially influence PH-YFP oligomerization.

**Figure 3:**
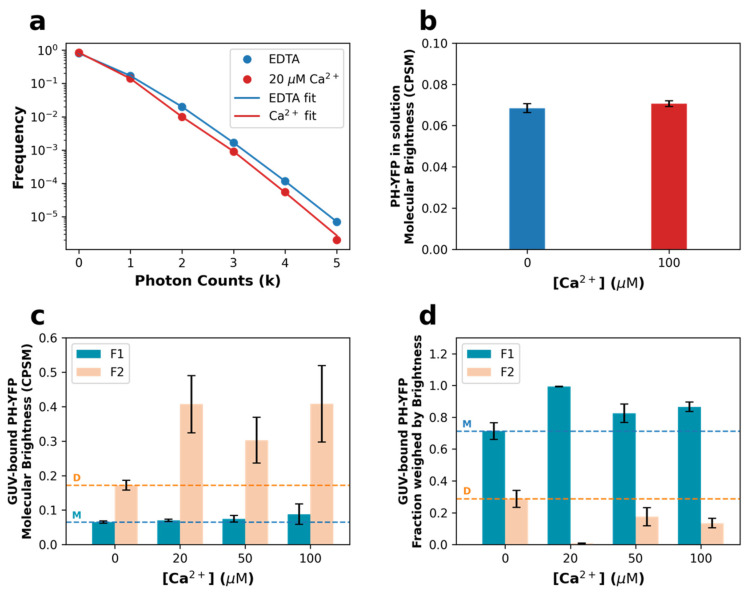
Disruption of PH-YFP oligomerization is dependent on Ca^2+^ concentration. (**a**) Representative photon counting histograms of PH-YFP in POPC:PI(4,5)P_2_ (95:5 molar ratio) GUVs in the presence (red) and absence (blue) of Ca^2+^. (**b**) Molecular brightness values recovered for PH-YFP in solution in the presence and absence of Ca^2+^. (**c**) Molecular brightness values and (**d**) fractions weighed by brightness recovered for PH-YFP in POPC:PI(4,5)P_2_ (95:5 molar ratio) GUVs in the presence and absence of Ca^2+^. Horizontal dashed lines represent the values obtained in the absence of Ca^2+^ for the monomer (M, orange) and dimer (D, blue) populations. Values represent means ± standard errors. Values from solution measurements for each condition are averages of four independent samples. Values from GUV measurements for each condition are averages of at least three different GUVs ($N_{0\;\mu M\;Ca^{2+}}=12,\;N_{20\;\mu M\;Ca^{2+}}=7,\;N_{50\;\mu M\;Ca^{2+}}=3,\;N_{100\;\mu M\;Ca^{2+}}=3$).

A proper quantitative assessment of the extent of PH-YFP oligomerization can be achieved through the use of photon counting histogram (PCH) analysis of the fluorescence fluctuation datasets used above for FCS, as PCH can resolve the presence of diffusing particles with different brightness [50,51,52,53]. If oligomerization occurs, at least two populations of fluorescent particles should be detected with distinct brightnesses. Analyzing the PCH for PH-YFP in solution, both sets of FFS data (in the presence and absence of Ca^2+^) were properly fitted by a single brightness population model. Furthermore, the brightness values confirmed that Ca^2+^ did not have a direct effect on PH-YFP oligomerization in solution, as there was no significant difference in the brightness recovered for the single population (Figure 3b), whether in the absence (ε = 0.068 ± 0.002 CPSM (brightness ± SEM)) or presence of Ca^2+^ (ε = 0.070 ± 0.001 CPSM) (*p* = 0.200 according to the Mann–Whitney U test).

However, the photon counting histograms obtained for PH-YFP bound to the membrane of GUVs could no longer be properly fitted by a single population model and were instead fitted with a two-brightness population model (see Supplementary Materials for details on the models). Representative photon counting histograms of PH-YFP bound to GUVs are shown in Figure 3a. Analyzing the brightness data recovered (Figure 3c), one of the populations presented a brightness value with negligible differences between the samples in the absence and presence of the several Ca^2+^ concentrations (ε_1_). This value was consistent with the value recovered for the protein in solution (Figure 3b) and, therefore, considered to represent the brightness of the monomeric (M) membrane-bound PH-YFP population (5mM EDTA: ε_1_ = 0.065 ± 0.003 CPSM; 20 μM Ca^2+^: ε_1_ = 0.071 ± 0.003 CPSM; 50 μM Ca^2+^: ε_1_ = 0.075 ± 0.010 CPSM; 100 μM Ca^2+^: ε_1_ = 0.088 ± 0.003 CPSM). Importantly, in the presence of Ca^2+^, this population was fully dominant, with the second population corresponding to less than 5% of particles and <12% of total fluorescence on average. This result confirms that the presence of Ca^2+^ inhibited PH-YFP oligomerization in the membrane. Upon total chelation of Ca^2+^ by EDTA, the second population, which we considered to be the oligomeric membrane-bound PH-YFP population, presented a higher brightness value (5 mM EDTA: ε2 = 0.172 ± 0.014 CPSM), consistent with a dimer (D), as it was 2.3 ± 0.3 times higher than the average of the values recovered for the lower brightness component. The fraction of dimers observed in the absence of Ca^2+^ corresponded to 28.7% ± 5.3% of total protein (Figure 3d). In the presence of Ca^2+^, as already discussed, the contribution of particles brighter than the monomer fell considerably, and the recovered brightness for the second component presented much higher uncertainty (20 μM Ca^2+^: ε2 = 0.407 ± 0.083 CPSM; 50 μM Ca^2+^: ε2 = 0.303 ± 0.066 CPSM; 100 μM Ca^2+^: ε2 = 0.408 ± 0.111 CPSM). These values are also higher than the brightness value obtained for the dimer in the presence of EDTA, possibly reflecting the presence of a small fractions of highly fluorescent, stable higher-order oligomers of PH-YFP, which are not affected by the presence of Ca^2+^, unlike dimers. Upon disruption of dimers by Ca^2+^, these oligomers which were already present became the only oligomeric population and, hence, the few particles observed presented higher brightness.

The fraction weighed by brightness can be used to quantify the total contribution of each population toward the total fluorescence collected. Here, we used it to more accurately quantify each population present on the surface of the GUVs (Figure 3d and Table 1). In the presence of Ca^2+^, the fraction of oligomerized population (F_2_) almost entirely disappeared at 20 μM Ca^2+^ (F_2_ = 0.7% ± 0.2%; *p* < 0.0001 according to the Mann–Whitney U test), when compared to the sample in the absence of Ca^2+^ (F_2_ = 28.7% ± 5.3%). On the other hand, at higher calcium concentrations (≥50 μM Ca^2+^), significant oligomerization took place, with fractions of oligomers comparable to the results obtained in the absence of calcium (Table 1).

The results obtained from FFS experiments regarding PH-YFP oligomerization at 20 and 100 µM calcium suggest a larger sensitivity to calcium levels than observed from homo-FRET measurements, where results obtained of 10 and 100 µM were comparable (Figure 1a). Given the differences in model membrane used, the lipid concentration was expected to have been far lower during FFS experiments (only some of lipids were hydrated during vesicle preparation) than the one employed in homo-FRET assays and, hence, the protein density was expected to have been significantly higher on the surface of GUVs than in LUVs. For the same reasons, the ratio of calcium ions and PI(4,5)P_2_ would also differ greatly between the two experiments. For the homo-FRET assays, this ratio was 2 and 20 for the different calcium concentrations, while, in FFS assays, these values were necessarily much larger due to the presence of lower PI(4,5)P_2_ concentration. This ratio is expected to influence PI(4,5)P_2_ cluster size and shape, as previously shown by molecular simulations [54]. In this way, the larger sensitivity to calcium concentrations observed by FFS could have been due to either increased protein density in GUVs or to differences in Ca^2+^:PI(4,5)P_2_ ratios.

The results presented above clearly demonstrate that Ca^2+^ has a dramatic inhibitory effect on the dimerization of PH-YFP, but a molecular mechanism that could explain this effect is not obvious. The fact that PH-YFP is shown to remain anchored to Ca^2+^-dependent PI(4,5)P_2_ clusters offers a possible explanation, as, in this case, interaction with a fairly small PI(4,5)P_2_ cluster could limit the range of protein-protein interactions available for the “trapped” protein. According to this model, in the absence of Ca^2+^-induced clustering of PI(4,5)P_2_, each PH-YFP domain after binding to the membrane is free to diffuse laterally within the membrane, and, in this two-dimensional environment where protein concentration is dramatically higher than in solution, protein–protein interactions are favored. On the other hand, in case of the formation of relatively small PI(4,5)P_2_ clusters (20 μM Ca^2+^), the membrane no longer presents a homogeneous environment for protein diffusion, since PI(4,5)P_2_ is segregated from the bulk lipids. In this case, proteins after binding to PI(4,5)P_2_ clusters smaller than the necessary to accommodate PH-YFP dimers (under 30 lipid molecules) are not totally free to diffuse laterally and become trapped or compartmentalized within these nanodomains. Additionally, if PI(4,5)P_2_ clusters exhibit electrostatic repulsion due to incomplete chelation, interaction between individual PI(4,5)P_2_ clusters could be rare, further reducing the probability of protein–protein interactions. These effects are likely to inhibit the protein–protein interactions seen in the absence of Ca^2+^. 

This rationalization is consistent with the observations that, according to FFS data, maximal inhibition of PH-YFP dimerization took place with 20 μM Ca^2+^, and that oligomerization recovered moderately for higher calcium levels. In fact, with increasing Ca^2+^ concentrations, cluster sizes are expected to increase [32,37] and could allow for the accommodation of a dimer within a single nanodomain. This results in a recovery of the oligomerized population (50 μM and 100 μM Ca^2+^). This proposed mechanism offers very interesting insight into how Ca^2+^ levels, through the formation of Ca^2+^-induced PI(4,5)P_2_ clusters, could possibly regulate protein–protein interactions between PI(4,5)P_2_-binding proteins and act as a regulatory step in signaling pathways.

## 4. Discussion

The interaction of divalent cations with PI(4,5)P_2_, especially Ca^2+^, has been the focus of several studies. Divalent cations have been found to interact strongly with PI(4,5)P_2_ and modulate its biophysical properties by influencing headgroup electrostatics via charge shielding [39], headgroup conformation [40], and PI(4,5)P_2_ lateral organization by promoting the formation of clusters [32,33]. In all, this implies that divalent cations and especially Ca^2+^, a common signal transduction element with a very buffered low physiological concentration, can have a strong role in the regulation of PI(4,5)P_2_. These findings have led us to study how Ca^2+^ directly influences the downstream protein–protein interactions of PI(4,5)P_2_-binding proteins. This is a crucial effect, as it is through binding proteins that PI(4,5)P_2_ carries out several of its important regulatory effects. In this work, we aimed to study how Ca^2+^-induced PI(4,5)P_2_ effects may influence the organization of PI(4,5)P_2_-binding proteins focusing on the effects on PH-YFP, a fusion protein that contains an isolated PLCδ1 PH domain linked to a YFP variant that is prone to dimerization.

Given the large structural changes from Ca^2+^-induced clustering, it makes sense that Ca^2+^ could influence membrane protein dynamics. We confirmed through FCS that PH-YFP membrane diffusion rates were slower in the presence of Ca^2+^, asserting that PH-YFP remained anchored to PI(4,5)P_2_ clusters and was unable to sequester the bound PI(4,5)P_2_ from these structures. This information is a strong indicator that cation-induced clusters might, indeed, be able to influence protein dynamics.

**Figure 4:**
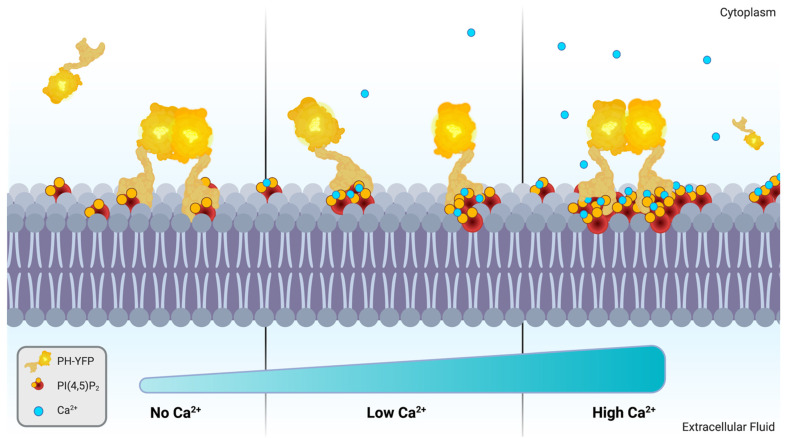
Schematic representation of Ca^2+^-dependent modulation of PH-YFP oligomerization through Ca^2+^-induced PI(4,5)P_2_ cluster size. Created with BioRender.com.

Since PH-YFP undergoes oligomerization in the membrane, we could also quantify how cation-induced PI(4,5)P_2_ clustering influenced protein–protein interactions for this specific protein model. We observed that Ca^2+^, in general, disrupted PH-YFP oligomerization in a reversible manner. Furthermore, this disruption was seen to be dependent on the concentration of Ca^2+^, which could be the result of increasing PI(4,5)P_2_ cluster sizes (Figure 4). In fact, an increase in cluster size is expected to increase the probability of incorporating multiple PH-YFP within the same PI(4,5)P_2_ nanodomain. In case these changes in PI(4,5)P_2_ organization occurred within plasma membranes in response to Ca^2+^ stimulus, it is possible that a multitude of different protein–protein interactions were dramatically impacted, as proteins were included or excluded from interaction by an on–off Ca^2+^ switch. Such a regulatory mechanism could act as an important step in signaling pathways, especially in the vicinity of Ca^2+^ channels, where more steep fluctuations of local Ca^2+^ concentrations occur before buffering of the divalent cation by Ca^2+^-binding proteins.

An important fact, which is not directly addressed in this article, is the influence of Mg^2+^ on PI(4,5)P_2_–protein interactions. While, in vivo, intracellular Ca^2+^ is maintained at very low levels (around 100 nM), with transient spikes in concentration (up to hundreds of μM), Mg^2+^ levels are kept at much higher and more stable concentrations. Free intracellular Mg^2+^ levels are typically around the 0.25–1 mM range. Although Mg^2+^ has a much weaker affinity for PI(4,5)P_2_ when compared to Ca^2+^, at 1 mM, it is able to induce PI(4,5)P_2_ clustering comparable to that observed in the Ca^2+^ range of concentrations studied here [36]. This could mean that PI(4,5)P_2_ is constitutively present in clusters and crosslinked with divalent cations. One also needs to consider that Ca^2+^ and Mg^2+^ will have a combined influence over PI(4,5)P_2_, which could buffer the effects caused by the fluctuation of Ca^2+^ levels. As the effect of Mg^2+^ has been often neglected, here included, it would be of interest for further studies to also emphasize its contribution.

Given that our results unequivocally associate the presence of divalent cations with considerable changes in PI(4,5)P_2_-binding protein oligomerization, critical re-evaluations of PI(4,5)P_2_–protein dynamics must be carried out for PI(4,5)P_2_–binding proteins that take these effects into account.

## Figures and Tables

**Table 1:** Fraction weighed by brightness values recovered with PCH analysis for PH-YFP in POPC:PI(4,5)P_2_ (95:5 molar ratio) GUVs in the presence and absence of Ca^2+^.

Ca^2+^ Concentration (μM)	F_1_ (Monomeric Population) (%)	F_2_ (Oligomeric Population) (%)
0	71.3 ± 5.3	28.7 ± 5.3
20	99.3 ± 0.2	0.7 ± 0.2
50	82.5 ± 5.7	17.5 ± 5.7
100	86.5 ± 3.0	13.5 ± 3.0

## Data Availability

Processed data used to obtain the figures published in this article can be found at https://github.com/Lp0lp/PIP_Prot_Impact (accessed on 11 May 2022). Unprocessed raw data can be obtained directly from the authors upon request.

## Supplementary Materials

The following supporting information can be downloaded at: https://www.mdpi.com/article/10.3390/biom12070912/s1, File S1: Supplementary materials and Methods [47,48,49,50,51,52,53]; Figure S1: Ca^2+^ does not inhibit membrane association of PH-YFP at the studied concentrations.
