# Supplementary material for: Impact of Ca2+-Induced PI(4,5)P2 Clusters on PH-YFP Organization and Protein-Protein Interactions
Source: Biomolecules. 2022 Jun 29;12(7):912. doi: 10.3390/biom12070912 (PMC9312469; doi:10.3390/biom12070912)
Supplement: Supplementary file 1 [file biomolecules-12-00912-s001.zip › biomolecules-1746590-supplementary.pdf]

Supplementary Material

Impact of Ca^{2+}-Induced $PI(4,5)P_2$ Clusters on PH-YFP Organization and Protein–Protein Interactions

Luís Borges-Araújo [1,2], Marina E. Monteiro [3], Dalila Mil-Homens [1,2], Nuno Bernardes [1,2], Maria J. Sarmento [3,4],
Ana Coutinho [1,2,5], Manuel Prieto [1,2] and Fábio Fernandes [1,2,6,*]

[1] IBB-Institute for Bioengineering and Biosciences, Instituto Superior Técnico, University of Lisbon,
1049-001 Lisbon, Portugal; lpborgesaraujo@tecnico.ulisboa.pt (L.B.-A.); dalilamil-homens@tecnico.ulisboa.pt (D.M.-H.); nuno.bernardes@tecnico.ulisboa.pt (N.B.); ana.coutinho@tecnico.ulisboa.pt (A.C.); manuel.prieto@tecnico.ulisboa.pt (M.P.)
[2] Associate Laboratory i4HB—Institute for Health and Bioeconomy at Instituto Superior Técnico, University of Lisbon, 1049-001 Lisbon, Portugal
[3] Centro de Química-Física Molecular and Institute of Nanoscience and Nanotechnology, Instituto Superior Técnico, University of Lisbon, 1049-001 Lisbon, Portugal; marina.monteiro@ist.utl.pt (M.E.M.); maria.sarmento@medicina.ulisboa.pt (M.J.S.)
[4] Instituto de Medicina Molecular, Faculty of Medicine, University of Lisbon, Avenida Prof. Egas Moniz, 1649-028 Lisbon, Portugal
[5] Departamento de Química e Bioquímica, Faculty of Sciences, University of Lisbon, 1749-016 Lisbon, Portugal
[6] Department of Bioengineering; Instituto Superior Técnico, University of Lisbon, 1049-001 Lisbon, Portugal
* Correspondence: fernandesf@tecnico.ulisboa.pt

Supplementary materials and Methods

Fluorescence correlation spectroscopy.

For FCS measurements performed in simple samples in solution, a model that takes into account three-dimensional translational diffusion and triplet state formation for *n* diffusing species was used[47–49]. Where $\boldsymbol{G(\tau)}$ is modelled by

$$G(\tau) = 1 + \frac{1}{N} \cdot \left(\frac{1 - T + T.e^{\frac{-\tau}{\tau_T}}}{1 - T} \right) \cdot \left\{ \sum_{i=1}^{n} \frac{f_i}{\left(1 + \frac{\tau}{\tau_D}\right)\sqrt{1 + \tau(S^{-2}\tau_D)}} \right\}, \tag{1}$$

Where the axial (ω_z) to lateral (ω_{xy}) dimension ratio, *S*, is given by $S = \frac{\omega_z}{\omega_{xy}}$, and the translational diffusion time, τ_D, is given by $\tau_D = \frac{\omega_{xy}^2}{4D}$. *N* corresponds to the average number of fluorescent particles in the observation volume, f_i is the fractional population of *n* different diffusion species, τ_T is the triplet lifetime and *T* corresponds to $\frac{f_t}{1 - f_t}$, where f_t is the fraction of fluorophores in the triplet state. For the modelling of data obtained from GUV membrane fluorescence, a model that considers two-dimensional translational diffusion through a Gaussian observation volume was used [50], described by

$$G(\tau) = \frac{1}{C\pi\omega_{xy}^2}\left(1 + \frac{4D\tau}{\omega_{xy}^2}\right)^{-1} = \frac{1}{N}\left(1 + \frac{\tau}{\tau_D}\right)^{-1}, \tag{2}$$

where, *C* corresponds to the particle concentration. The dimensions of the focal volume were determined by calibration with rhodamine 110 in milliQ water using the same optical setup as the samples. A rhodamine 110 diffusion coefficient (D) of 440 $\mu m^2 s^{-1}$ was considered [51]. Analysis of all the FCS experimental data was carried out using the ISS Vista software. This program uses a

Marquardt–Levenberg nonlinear least-squares fitting routine and the goodness of the fittings can be judged by the recovered χ^2 value and the random distribution of the weighted residuals.

Photon counting histogram.

Recorded fluorescence fluctuation data can be used to generate photon counting histograms that access information on the molecular brightness, $\boldsymbol{\varepsilon}$, and average number of particles. To describe the PCH of a freely diffusing species, we must first consider the case of a single, diffusing particle enclosed in a small volume V_0, where over a long enough observation time, the probability of the particle appearing at any position within V_0 will be the same. Combining the appropriate probability distribution of particle diffusion and PSF with Mandel's Formula, that describes the photon counting statistics for the semiclassical model of photon detection, we obtain the probability of detecting $k > 0$ photons for one fluorescent particle in $\boldsymbol{V_0}$ [52,53].

$$\boldsymbol{p^{(1)}(k;V_0,\varepsilon) = \frac{1}{V_0}\int Poisson\,[k,\varepsilon\cdot W(\overrightarrow{r})]d\vec{r}\ ;\ Poisson(k,\lambda) = \frac{\lambda^k e^{-\lambda}}{k!}}, \tag{3}$$

This equation describes the PCH of a single diffusing fluorescent particle. It is the weighted average of Poissonian distributions for k counts. $\boldsymbol{W(\overrightarrow{r})}$ is the function that describes the observation volume profile, considers excitation laser intensity and the detection efficiency as a function of the particle position. $\boldsymbol{\varepsilon}$ is the molecular brightness where:

$$\boldsymbol{\varepsilon = I_0^n \beta \eta_W T_s}, \tag{4}$$

The molecular brightness characterizes the average number of detected photons per sampling time ($\boldsymbol{T_s}$) per molecule. Through the parameter $\boldsymbol{\beta}$, the molecular brightness takes into account the particle fluorescence quantum yield, extinction coefficient, and all of the instrument-dependent factors. The ratio $\boldsymbol{\varepsilon_{sec} = \varepsilon/T_s}$ is used as it allows a more convenient comparison between experiments. $\boldsymbol{\varepsilon_{sec}}$ is expressed in counts per second per molecule (cpsm) [53]. After defining the PCH for a single diffusing particle, we then consider the possibility of finding N identical but independent particles that diffuse within V_0. The distribution of the photon counts (k), will then be given by the Nth convolution of the distribution defined for a single particle, p, with itself:

$$\boldsymbol{p^{(N)}(k;V_0,\varepsilon) = (p^{(1)}\otimes p^{(1)}\otimes \ldots \otimes p^{(1)})(k;V_0,\varepsilon)}, \tag{5}$$

The case where no particles are present in the reference volume and no photons are detected is also considered

$$\boldsymbol{p^{(0)}(k;V_0,\varepsilon) = \begin{cases} 1, k = 0 \\ 0, k \neq 0 \end{cases}}, \tag{6}$$

If we account for the fluctuation of the number of particles N inside V_0, given a certain concentration, c, then the probability of finding N particles in V_0 follows a Poisson distribution with the mean value cV_0. However, while c is adequate to characterize the concentration of particles, it is more straightforward to use the average of number of particles, $\boldsymbol{\overline{N}}$, in a certain volume V, as a fitting parameter. In this way, the PCH for a single species is given by:

$$\boldsymbol{P(k;\,\overline{N},\varepsilon) = \sum_{N=0}^{\infty} p^{(N)}(k;Q,\varepsilon)\cdot Poisson(N,Q\overline{N})}, \tag{7}$$

$$\boldsymbol{Q = \frac{V_0}{V}}, \tag{8}$$

The volume ratio Q serves as a numerical simplification, cancelling the units of volume in the PCH integration, as $\boldsymbol{p}^{(N)}(\boldsymbol{k};\boldsymbol{V_0},\boldsymbol{\varepsilon})$ is independent from the choice of V_0 as long as it is large enough to obtain a positive probability of zero photon counts [52,53]. In all our data analysis we arbitrarily chose Q = 6. To account for deviations of the experimental observation volume from the 3D Gaussian approximation, Huang et al. [52] implemented a correction that takes into consideration the contribution to the PCH integral from the out-of-focus region. This correction introduced a new parameter, *F*, that accounts for the fraction of detected photons from the non-Gaussian part of the observation volume. In this way, after the correction of the 3D Gaussian approximation, the PCH model for a confocal microscope with one-photon excitation is described by the following equations:

$$\boldsymbol{P}(\mathbf{1};\boldsymbol{Q},\boldsymbol{\varepsilon}) = \frac{1}{(1+F)^2}\left[\boldsymbol{p}_G\,(\mathbf{1};\boldsymbol{Q},\boldsymbol{\varepsilon}) + \frac{\varepsilon F}{2\sqrt{2Q}}\right], \tag{9}$$

$$\boldsymbol{P}(\boldsymbol{k};\boldsymbol{Q},\boldsymbol{\varepsilon}) = \frac{1}{(1+F)^2}\boldsymbol{p}_G\,(\boldsymbol{k};\boldsymbol{Q},\boldsymbol{\varepsilon})\ \textbf{for}\ \boldsymbol{k} > \mathbf{1}, \tag{10}$$

and,

$$\boldsymbol{P}(\mathbf{0};\boldsymbol{Q},\boldsymbol{\varepsilon}) = \mathbf{1} - \sum_{k}^{\infty}\boldsymbol{p}\quad(\boldsymbol{k};\boldsymbol{Q},\boldsymbol{\varepsilon}), \tag{11}$$

where,

$$\boldsymbol{p}_G\,(\boldsymbol{k};\boldsymbol{Q},\boldsymbol{\varepsilon}) = \frac{1}{Q\pi^{1/2}k!}\int_0^{\infty}\boldsymbol{\gamma}\left(\boldsymbol{k},\boldsymbol{\varepsilon}e^{-2x^2}\right)\boldsymbol{dx}, \tag{12}$$

$\boldsymbol{p}_G$ is the analytical expression for the PCH integration using a 3D Gaussian approximation for one-photon excitation and confocal detection and γ is the incomplete Gamma function. For more than two species, differences in photophysical properties, like quantum yield, are considered and included in the coefficient $\boldsymbol{\varepsilon}$ that will be different for each species. This allows us to detect and quantify the presence of different species with different brightness, such as monomers and oligomers in a sample. In the case of multiple independent species with different brightness, the photon counting histogram can be obtained by convoluting the photon counting histograms of the individual species:

$$\boldsymbol{P}(\boldsymbol{k};\ \boldsymbol{N}\mathbf{1},\ \boldsymbol{\varepsilon}\mathbf{1},\boldsymbol{N}\mathbf{2},\ \boldsymbol{\varepsilon}\mathbf{2}\ldots\boldsymbol{Nn},\ \boldsymbol{\varepsilon n}) = \boldsymbol{P}(\boldsymbol{k};\ \boldsymbol{N}\mathbf{1},\ \boldsymbol{\varepsilon}\mathbf{1})\otimes\boldsymbol{Pk};\ \boldsymbol{N}\mathbf{2},\ \boldsymbol{\varepsilon}\mathbf{2}\otimes\ldots\otimes\boldsymbol{P}(\boldsymbol{k};\ \boldsymbol{Nn},\ \boldsymbol{\varepsilon n}), \tag{13}$$

For PH-YFP samples, these equations were globally fitted to the experimental data using a homemade MATLAB implementation by considering the presence of two populations with different brightness, ε_1 and ε_2, that correspond to the monomeric and oligomerized form of the protein, respectively. In this analysis, we consider that the monomer brightness does not change from sample to sample by linking ε_1 across all the curves obtained. The correction factor, *F*, can be determined by a calibration procedure using 5 nM of rhodamine 110 and subsequently fixed for the other samples measured in the same chamber slide.

Using the brightness and number of particles, obtained through PCH analysis of each data set, we calculated the fraction weighed by brightness ($\boldsymbol{F_i}$), given by the following equation:

$$\boldsymbol{F_i} = \frac{N_i\varepsilon_i}{\sum_1^i N_i\varepsilon_i}, \tag{14}$$

where, $\boldsymbol{N_i}$ and $\boldsymbol{\varepsilon_i}$ refer to the number of particles and brightness, respectively, of population $\boldsymbol{i}$. The fraction weighed by brightness can be used to quantify the total contribution of each population towards the total fluorescence collected. Here, we will use it to quantify each population of fluorescent proteins more accurately.

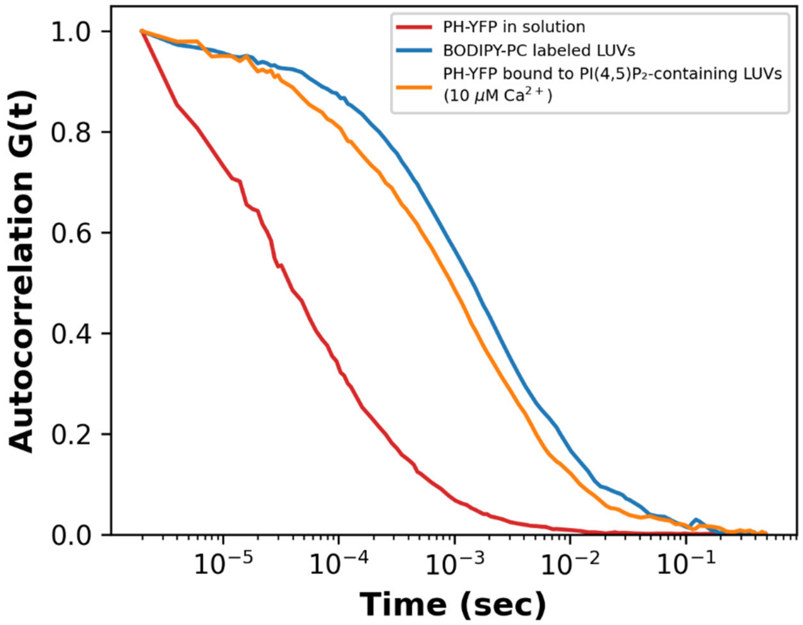

Figure S1. Ca^{2+} does not inhibit membrane association of PH-YFP at the studied concentrations. FCS autocorrelation curves obtained for PH-YFP in solution (red), 100 μM BODIPY-PC labelled LUVs (blue) and PH-YFP bound to 100 μM of POPC:PI(4,5)P_2 (95:5 molar ratio) LUVs, in the presence of 10 μM Ca^{2+} (blue). Autocorrelation curves for PH-YFP in the presence of POPC:PI(4,5)P_2 liposomes and calcium can be fitted with a single component of diffusion coefficient identical to the one recovered for labeled vesicles.
